# Compartment syndrome as a novel complication of extended spectrum beta lactamase *Escherichia coli* necrotising soft tissue infection – A case report

**DOI:** 10.1016/j.ijscr.2022.107574

**Published:** 2022-08-31

**Authors:** Damien Gibson, Oliver Chow, Ishith Seth, Adrian Hang Yue Siu, Johnny Kwei

**Affiliations:** a Department of Surgery, St George Hospital, Sydney, NSW, Australia; b School of Medicine, The University of New South Wales, Sydney, Australia; c Macquarie University Hospital, Plastics and Reconstructive Surgery, Macquarie Park, NSW, Australia; d Bendigo and Northern District Base Hospital, Surgery, Bendigo, VIC, Australia; e Department of Surgery, Bankstown Hospital, Sydney, NSW, Australia; f Northern Beaches Hospital, Plastic Surgery, Frenchs Forest, NSW, Australia

**Keywords:** NSTI, necrotising soft tissue infections, ESBL, extended spectrum beta lactamase, MRSA, methicillin-resistant *Staphylococcus aureus*, IVIG, intravenous immunoglobulin, HBOT, hyperbaric oxygen therapy, Soft tissue infections, Compartment syndromes, Fasciitis, Necrotising, Case report

## Abstract

**Introduction and importance:**

Necrotising soft tissue infections (NSTI) encompass a group of destructive soft tissue disease processes which can involve skin, subcutaneous tissue, fascia and or muscle, associated with rapid spread along tissue planes and mortality. Clinical presentations include progressive pain, suppuration/necrosis and systemic toxicity with haemodynamic instability. While diagnosis is based on clinical findings it can be augmented with imaging. Treatment is typically in the form of resuscitation, immediate administration of broad spectrum intravenous antibiotics and urgent source control through radical surgical debridement.

**Case presentation:**

An 82-year-old man presented with left forearm/hand pain and fevers in the context of immunocompromise. Examination found tense swelling of the left volar and dorsal forearm and hand, absent distal pulses with pain and paraesthesia over both surfaces. He underwent surgical debridement with fasciotomy and remained in intensive care with blood cultures revealing ESBL *E. coli*.

**Clinical discussion:**

Compartment syndrome is a rare complication of NSTI and its clinical presentation can obscure early diagnosis. ESBL *E. coli* is an uncommon pathogen to cause monomicrobial infection and must be accounted for when considering broad spectrum empirical antibiotic cover.

**Conclusion:**

Review of this case and the literature show a rare presentation of NSTI and highlights the importance of early diagnosis based on even a small index of suspicion. It also shows the key significance rationalisation of antibiotics as soon as practicable, given that even broad spectrum empirical cover can be inappropriate in the context of novel microorganisms, particularly in high risk patients.

## Highlights

• First recorded case of monomicrobial ESBL *E. coli* compartment syndrome
• ESBL *E. coli* NSTI is rare and not covered by many empirical antimicrobial guidelines.
• Compartment syndrome secondary to NSTI is uncommon and associated with streptococci.
• Early recognition and prompt management are essential to prognosis.

## 1 Introduction

Necrotising soft tissue infections (NSTI) encompass a group of destructive soft tissue disease processes which can involve skin, subcutaneous tissue, fascia and/or muscle, with rapid dissemination though tissue planes and considerable mortality [1]. Risk factors for NSTI include penetrating trauma, recent surgery, immunosuppression, obesity, malignancy, cirrhosis and pregnancy [1], [2]. Clinical presentations include pain, suppuration, necrosis with associated systemic toxicity and haemodynamic instability [1]. While diagnosis is largely clinical, it can be augmented with X-ray or computer tomography (CT) to identify gas within soft tissue planes, asymmetrical fascial thickening and fat stranding – though imaging should not delay management [1]. Treatment is in the form of resuscitation, immediate administration of broad spectrum intravenous antibiotics and urgent source control through radical surgical debridement [1]. This case has been reported in line with the SCARE 2020 criteria and PROCESS guidelines [3], [4].

## 2 Case description

An 82-year-old man presented with left forearm/hand pain and fevers in the context of immunocompromise secondary to stage IV Hodgkin's lymphoma and recent cycle of cyclophosphamide, hydroxydaunorubicin, oncovin and prednisone (CHOP) chemotherapy regime. He had a cannula inserted into the volar forearm one week earlier for administration of chemotherapy and developed worsening symptoms over 10 days. On examination there was mild cellulitis, with some bruising and discolouration similar in nature to other areas of his skin. The key finding was tense swelling of the left volar and dorsal forearm and hand, associated with pain on passive stretch and paraesthesia over both surfaces. The tense swelling was only marked when compared to the wasted right arm. There was also delayed capillary refill of over 3 s with radial and ulnar pulses not present. A clinical picture of compartment syndrome became apparent.

While systemically well upon triage, awaiting review he became unstable in the emergency department with signs of septic shock. He was commenced on piperacillin/tazobactam, vancomycin and clindamycin as per local guidelines. Plastic surgical input was sought. The constellation of fevers and raised inflammatory markers with no overt fracture or bleeding, made NSTI the most likely diagnosis prompting immediate operative intervention.

Urgent fasciotomy of the dorsal, volar forearm and dorsal interossei compartment release was performed by the consultant plastic surgeon assisted by his senior registrar in a major local hospital. This was followed by aggressive debridement of devitalised tissue of the forearm to healthy bleeding tissue. Operative findings included rapid development of superficial pustule formation, ‘dishwasher’ fluid in the suprafascial plane (dorsal and volar) and tense bulging flexor and extensor muscle compartments. Post fasciotomy, distal capillary refill returned to less than 3 s and pulses were felt at the wrist. The early identification of compartment syndrome and urgent fasciotomy resulted in limb salvage for this patient.

He remained unstable in ICU post debridement requiring vasopressors until antibiotic rationalisation from piperacillin/tazobactam to meropenem based on culture findings of extended spectrum beta lactamase *Escherichia coli* (ESBL *E. coli*). The patient required daily dressing changes with ongoing intravenous antibiotics for a number of weeks. The patient experience was painful, with daily dressing changes often performed under nitrous or anaesthetic in intensive care. He returned to the operating theatre for reconstruction of his debridement and fasciotomies wounds three weeks after initial presentation. One week later he was transferred to a rehabilitation facility for hand and occupational therapy.

## 3 Discussion

This case an example of an atypical NTSI presentation, with compartment syndrome and culture isolates of monomicrobial ESBL *E. coli*, a phenomenon which has not previously been described in the literature. While compartment syndrome from streptococcal sources is uncommonly described in the literature, there are no reports of *E. coli* NTSI compartment syndrome [5]. Although uncommon, the prevalence of monomicrobial NTSI *E. coli* is increasing at a global rate of 1.1 % annually and makes up 10 % NTSI infections in Australia and New Zealand [5], [6].

Only 14 case reports of non-traumatic compartment syndrome secondary to Group A β-haemolytic streptococci have been noted in the literature [5], [7]. The mechanism of this novel complication involves the Lipid A component of the *E. coli* endotoxin, which stimulates cytokine production (with associated toxaemia), complement activation and coagulation cascade activation resulting in acute disseminated intravascular coagulation and platelet and clotting factor depletion [3].

The Australian Therapeutic Guidelines state empirical antibiotic coverage should include gram positive, gram negative and anaerobic bacteria: this is typically in the form of some combination of a beta lactam/beta lactamase inhibitor (such as piperacillin/tazobactam or a carbapenem such as meropenem) with an agent to address MRSA (such as vancoymcin) and anaerobic organisms (such as lincomycin or clindamycin) [5], [6]. Clindamycin therapy should continue until culture results return due to its suppression of Group A streptococcus virulence proteins via opsonisation and evidence suggesting its inclusion in treatment can reduce mortality [9]. The antibiotic regime should be rationalised against stain and culture results as soon as practicable. This case of ESBL *E. coli* represents a rare entity which would not be covered through use of many antimicrobial guidelines for NSTI.

Adjunctive treatments have been proposed in the literature: namely intravenous immunoglobulin (IVIG) and hyperbaric oxygen therapy (HBOT) [1], [8]. IVIG is largely implicated for use against Group A streptococcus infections through antigen neutralisation and inflammatory response modulation [1]. HBOT has been suggested to reduce local oedema, increase angiogenesis and may have lethal effects on anaerobic bacteria although the evidence is conflicting [10].

## 4 Conclusion

This case reveals an atypical presentation of NSTI and highlights the importance of early diagnosis based on even a small index of suspicion. Without early identification with prompt surgical management, critical limb ischemia and sepsis would have resulted in considerable morbidity and likely mortality. This case has changed our practice, with increasing prevalence of multidrug resistant organisms our preference between piperacillin/tazobactam and a carbapenem now favours a carbapenem especially in the immunocompromised. It also shows the significance of antibiotic rationalisation as soon as practicable, given that even broad spectrum empirical cover can be inappropriate in the context of novel microorganisms, particularly in high-risk immunocompromised patients.

## Provenance and peer review

Not commissioned, externally peer-reviewed.

## Consent

Written informed consent was obtained from the patient for publication of this case report and accompanying images. A copy of the written consent is available for review by the Editor-in-Chief of this journal on request.

## Ethical approval

Nil.

## Funding

Nil.

## Guarantor

Dr Damien Gibson.

## Research registration number

1. Name of the registry: Nil
2. Unique identifying number or registration ID: Nil
3. Hyperlink to your specific registration (must be publicly accessible and will be checked): Nil.

## CRediT authorship contribution statement

Damien Gibson (Conceptualization; Data curation; Formal analysis; Writing - original draft; Writing - review & editing), Oliver Chow (Formal analysis; Visualization; Writing original draft; Writing - review & editing), Ishith Seth (Writing - review & editing), Adrian Hang Yue Siu (Writing - review & editing), Johnny Kwei (Supervision; Conceptualization; Project administration).

## Declaration of competing interest

None to declare.

## References

[1] Stevens D.L., Bryant A.E. (2017). Necrotizing soft-tissue infections. N. Engl. J. Med..

[2] Hakkarainen T.W., Kopari N.M., Pham T.N., Evans H.L. (2014). Necrotizing soft tissue infections: review and current concepts in treatment, systems of care, and outcomes. Curr. Probl. Surg..

[3] Agha R.A., Borrelli M.R., Farwana R., Koshy K., Fowler A., Orgill D.P., SCARE Group (2018). The PROCESS 2018 statement: updating consensus Preferred Reporting Of CasE Series in Surgery (PROCESS) guidelines. Int. J. Surg..

[4] Agha R.A., Franchi T., Sohrabi C., Mathew G., for the SCARE Group (2020). The SCARE 2020 guideline: updating consensus Surgical CAse REport (SCARE) guidelines. Int. J. Surg..

[5] Kleshinski J., Bittar S., Duggan J.M., Wahlquist M., Ebraheim N. (2008). Review of compartment syndrome due to group a streptococcal infection. Am. J. Med. Sci..

[6] Dhanasekara C.S., Marschke B., Morris E., Kahathuduwa C.N., Dissanaike S. (2021). Global patterns of necrotizing soft tissue infections: a systematic review and meta-analysis. Surgery.

[7] Wong K., Nicholson D.J., Gray R. (2005). Lower limb compartment syndrome arising from fulminant streptococcal sepsis. ANZ J. Surg..

[8] Stevens D.L., Bisno A.L., Chambers H.F., Everett E.D., Dellinger P., Goldstein E.J. (2005). Practice guidelines for the diagnosis and management of skin and soft-tissue infections. Clin. Infect. Dis..

[9] (2021 Mar). Therapeutic Guidelines [digital].

[10] Young M.H., Engleberg N.C., Mulla Z.D., Aronoff D.M. (2006). Therapies for necrotising fasciitis. Expert. Opin. Biol. Ther..

